# Comparison of muscle activity distribution in the biceps brachii using monopolar and bipolar multichannel surface electromyography

**DOI:** 10.1371/journal.pone.0325464

**Published:** 2025-07-07

**Authors:** Kakeru Ito, Akio Kikuchi, Toshiaki Sato

**Affiliations:** 1 Graduate School of Health Sciences, Yamagata Prefectural University of Health Sciences, Yamagata, Japan; 2 Department of Occupational therapy, Faculty of Health Sciences, Yamagata Prefectural University of Health Sciences, Yamagata, Japan; Fondazione Policlinico Universitario Gemelli IRCCS, ITALY

## Abstract

The detailed distribution of muscle activity within the biceps brachii (BB) muscle during isometric contraction as contraction intensity increases is not yet fully understood using multichannel surface electromyography (EMG). This study aimed to elucidate the distribution of muscle activity within the BB muscle during isometric contractions using monopolar and bipolar recording methods with multichannel surface EMG. The participants were 23 healthy young adults who performed a gradually increasing load task involving right elbow flexion for 10 seconds. The results showed that muscle activity was higher in the proximal medial area from the middle region with the monopolar recording method, and higher in the central medial area with the bipolar recording method as contraction intensity increased. These results with the monopolar recording method were consistent with previous reports regarding the increased shortening rate of the BB muscle from the middle to the proximal area and the migration of the innervation zone. Therefore, the monopolar recording method of multichannel surface EMG is considered more suitable for clarifying the detailed distribution of muscle activity within the same muscle.

## Introduction

Needle and surface electromyography (EMG) have been widely used to measure muscle activity. Needle EMG is an accurate but invasive recording method because electrodes are inserted directly into muscle tissue. On the other hand, surface EMG is a simple and non-invasive method as it involves attaching electrodes to the skin surface. In conventional surface EMG, electrodes are typically placed a few centimeters apart near the center of the muscle belly to measure muscle activity. However, signals detected by a single pair of electrodes provide only limited information about the muscle [1]. Even when multiple electrodes are used, their relatively large size makes it difficult to capture detailed activity distribution within the same muscle.

Recently, multichannel surface EMG systems, which combine over 60 electrodes into a single array has been developed. When applied to the rectus femoris during hip flexion exercises, higher muscle activity has been observed in the proximal region during strong contractions [2]. Studies of weak contractions in the biceps brachii (BB) muscle have shown higher muscle activity in the medial region [3]. Thus, multichannel surface EMG enables detailed comparison of muscle activity distribution within the same muscle. Most studies examining muscle activity distribution within a single muscle have used the bipolar recording method for data collection and analysis [2–3]. This method removes common mode signals from each electrode, reducing noise generated by skin-electrode attachment through signal cancellation [4–6]. However, bipolar recording method may exclude common EMG signals along with noise during the cancellation process [6–7], potentially omitting relevant muscle information [6,8]. In contrast, the monopolar recording method involves attaching reference electrodes to electrically inactive areas, such as bones, to record signals. Although this method is more sensitive to noise, it captures all EMG signals from the measured muscle without the influence of signal removal [4,7,8].

Although numerous studies have investigated the acquisition and interpretation of signals using high-density surface electromyography (HDEMG), direct comparisons between monopolar and bipolar electrode configurations in the context of spatial analysis of muscle activity remain limited [9–10]. While the CEDE consensus offers comprehensive recommendations regarding HDEMG matrix design [11], it does not explicitly address how different recording configurations may influence the spatial characteristics of EMG signals. This suggests that further research is warranted to clarify the effects of electrode configuration on spatial EMG interpretation.

Accordingly, the present study aims to elucidate the influence of monopolar and bipolar electrode configurations on the spatial interpretation of muscle activation using multichannel surface EMG. Specifically, we examined the distribution of BB muscle activity across varying contraction intensities to determine which recording method provides a more accurate representation of intramuscular activation patterns.

## Methods

### Participants

The participants were 23 healthy young adults (8 males and 15 females) with no pain or injury in their limbs or trunk and no history of orthopedic or neurological diseases. Their basic characteristics were as follows: age 21.7 ± 0.9 years, height 163.6 ± 7.6 cm, weight 56.3 ± 9.4 kg, and all were right-handed. The study was conducted from December 2023 to April 2024. All participants provided written informed consent, and the study adhered to the ethical standards set by the Declaration of Helsinki. This study was approved by the Ethics Committee of Yamagata Prefectural University of Health Sciences (No. 2311−24).

### Experimental design

**Fig 1:**
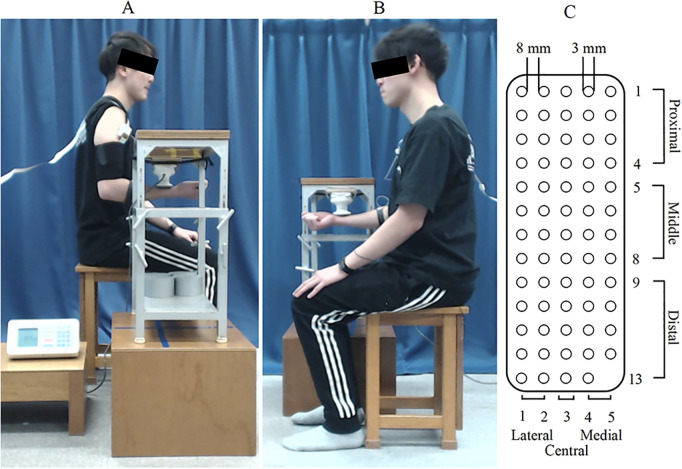
Experimental posture and matrix electrodes. **(A) (B)** The experimental posture involved sitting with the upper arm at the side of the body, the elbow flexed at 90°, and the forearm supinated at 90°. A and B showed the right and left side view, respectively. **(C)** The matrix electrodes were 3 mm in diameter, arranged in 5 columns and 13 rows with an 8 mm interelectrode distance, totaling 64 electrodes due to the absence of one electrode in the bottom-right corner. Regions were compared as follows: proximal, middle, and distal, each consisting of 4-5 rows from the top; and lateral, central, and medial, each consisting of 1-2 columns from the outer side.

All participants performed isometric voluntary contractions of the right elbow flexion with progressively increasing loads. The posture involved a seated position with the upper arm kept close to the body, the elbow flexed at 90°, and the forearm supinated at 90° (Fig 1A). First, the participants performed two maximal isometric contractions using a manual muscle force meter (manual muscle force sensor EG-230, Sakai Medical, Tokyo, Japan). Maximal isometric contractions were performed for 5 seconds with verbal encouragement. The higher value of the two measurements was considered the muscle force at maximum voluntary contraction (MVC). The manual muscle force meter was attached to a table with weights and adjusted to contact the distal forearm (below the radial styloid process) perpendicularly (Figs 1A and 1B). The task was performed three times with visual feedback provided on a monitor to achieve 0–100% MVC in 10 seconds. Participants practiced before each measurement and took sufficient rest between trials. If these criteria were met, the task was repeated after providing sufficient rest. The trial showing the most linear increase in muscle output was selected for analysis [12].

### EMG recording

Surface EMG was recorded from the right BB muscle using a matrix electrode (GR08MM1305, OT Bioelettronica, Italy) consisting of 64 electrodes arranged in a two-dimensional planar array. Each electrode was 3 mm in diameter and 8 mm apart in a 5-column by 13-row configuration (Fig 1C). The skin was prepared with abrasive paste and alcohol prior to electrode application. The center of the electrode array aligned with the distal third of the line connecting the acromion and the cubital fossa. The electrodes were attached along the direction of the muscle fibers and secured with a belt. The strap on the left wrist served as the reference, while the strap on the right forearm was connected to the adapter for the matrix electrode to amplify the signal and reduce noise (Figs 1A and 1B). Monopolar surface EMG signals were recorded using a 16-bit AD converter (Quattrocento, OT Bioelettronica, Italy) with a sampling frequency of 2048 Hz and a band-pass filter of 10–500 Hz. Bipolar surface EMG signals were derived by differencing the monopolar surface EMG signals between adjacent electrodes along the longitudinal axis, resulting in a total of 59 signals. EMG signal recording and analysis were performed using OTBioLab+ Version 1.5.5. software (OTBioLab + , OT Bioelettronica, Italy).

### Data processing

The average rectified value (ARV) was calculated every second for each recording method. The average value at 10 seconds of maximum contraction intensity was set as 100%, and normalized %ARV was calculated and used for each time point [12]. Additionally, ARV maps were created to visualize regional differences by converting ARV values to a color scale. For quantitative comparison of regional differences, the electrodes were divided into proximal, middle, and distal regions (4–5 rows each) from top to bottom, and into lateral, central, and medial regions (1–2 columns each) from outside to inside (Fig 1C). %ARV for each region was calculated every second.

### Statistical analysis

First, a two-way repeated measures ANOVA was conducted to determine whether regional differences in %ARV changes were significant with progressive increase in muscle output both the monopolar and bipolar recording methods. Next, normality was assessed using the Shapiro-Wilk test, and regional differences at each time point were examined using repeated measures ANOVA for normally distributed data and Friedman’s test for non-normally distributed data. If significant main effects were found in these tests, post-hoc multiple comparisons were conducted. Additionally, for comparisons of overall %ARV recording methods, paired t-tests were used for data confirmed to be normally distributed by the Shapiro-Wilk test, while Wilcoxon signed-rank tests were applied to non-normal data. Since the participants included both males and females, the influence of gender differences on muscle output and activity was examined by comparing %MVC and %ARV of each recording method. We performed Shapiro-Wilk tests to assess normality for %MVC and %ARV. For normally distributed data, t-tests were conducted, while Mann-Whitney U tests were used for non-normally distributed data. To address the issue of multiple comparisons, the Bonferroni method was applied to adjust the significance level. Statistical analysis was performed using R Version 4.3.3, with a significance level set at 5%.

## Results

Of the 23 participants who performed the task, 18 participants (7 males and 11 females) were included in the analysis, excluding a total of 5 participants: 2 participants were excluded due to trunk movement during the task and 3 participants were excluded due to noise abnormalities during the measurement. The average isometric elbow flexion strength at MVC, measured with the manual muscle force meter, was 119.1 ± 34.0 N (Table 1).

**Table 1:** Participants information.

	Male (n = 7)	Female (n = 11)	All (n = 18)
**Height (cm)**	171.1 (6.4)	160.1 (5.5)	164.4 (7.9)
**Weight (kg)**	65.7 (10.1)	51.5 (4.6)	57.1 (10.0)
**Age (year)**	21.4 (1.0)	21.6 (0.8)	21.6 (0.9)
**MVC (N)**	131.3 (37.2)	111.3 (28.9)	119.1 (34.0)
**%MVC**			
** 1s**	6.8 (4.6)	5.4 (2.1)	5.9 (6.6)
** 2s**	14.8 (3.0)	13.8 (4.4)	14.2 (3.9)
** 3s**	25.3 (5.6)	21.5 (5.3)	23.0 (5.7)
** 4s**	31.7 (3.2)	30.4 (6.1)	30.9 (5.2)
** 5s**	41.5 (4.9)	39.7 (4.9)	40.4 (5.0)
** 6s**	51.4 (5.3)	50.4 (6.8)	50.8 (6.3)
** 7s**	64.5 (6.7)	60.6 (6.0)	62.1 (6.6)
** 8s**	70.4 (4.9)	71.0 (6.4)	70.8 (5.8)
** 9s**	80.0 (6.4)	80.6 (5.6)	80.4 (5.9)
** 10s**	86.3 (7.6)	86.2 (6.0)	86.3 (6.6)
**%ARV- monopolar**			
** 1s**	22.7 (6.1)	19.2 (5.6)	20.5 (6.0)
** 2s**	31.7 (7.4)	23.7 (7.2)	26.8 (8.2)
** 3s**	37.5 (10.1)	29.2 (6.0)	32.4 (8.8)
** 4s**	45.8 (9.4)	37.2 (9.0)	40.5 (10.1)
** 5s**	57.4 (10.5)	46.3 (7.9)	50.6 (10.5)
** 6s**	69.4 (9.8)	55.3 (9.1)	60.8 (11.6)
** 7s**	73.0 (9.3)	69.5 (11.5)	70.9 (10.9)
** 8s**	79.8 (8.6)	83.9 (16.2)	82.3 (13.9)
** 9s**	94.3 (9.1)	98.0 (16.1)	96.6 (13.9)
** 10s**	100.0 (0)	100.0 (0)	100.0 (0)
**%ARV- bipolar**			
** 1s**	27.4 (7.2)	23.4 (8.7)	25.0 (8.4)
** 2s**	31.0 (6.2)	25.6 (8.9)	27.7 (8.4)
** 3s**	37.2 (9.1)	29.1 (7.5)	32.2 (9.1)
** 4s**	42.7 (9.9)	34.6 (8.8)	37.8 (10.1)
** 5s**	49.8 (7.0)	42.0 (7.5)	45.0 (8.3)
** 6s**	59.3 (3.7)	51.4 (10.6)	54.5 (9.4)
** 7s**	70.3 (7.6)	65.1 (14.6)	67.1 (12.6)
** 8s**	75.8 (6.4)	80.0 (18.8)	78.3 (15.4)
** 9s**	91.2 (5.7)	96.3 (15.1)	94.3 (12.5)
** 10s**	100.0 (0)	100.0 (0)	100.0 (0)

Mean (Standard deviation).

MVC: maximum voluntary contraction.

ARV: average rectified value.

**Fig 2:**
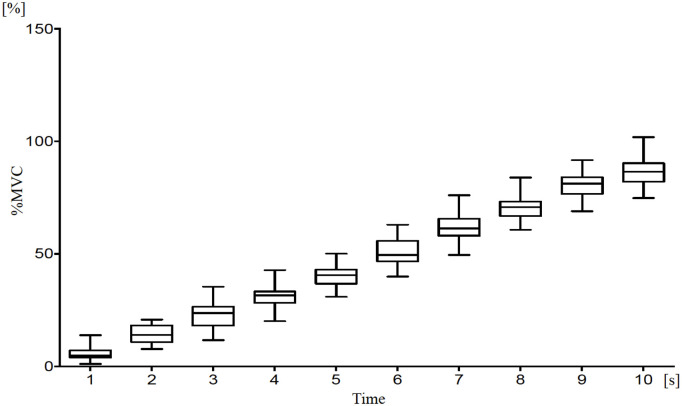
Temporal changes in % maximum voluntary contraction (MVC) over a 10-second period using multichannel surface electromyography. The %MVC increased over time.

**Fig 3:**
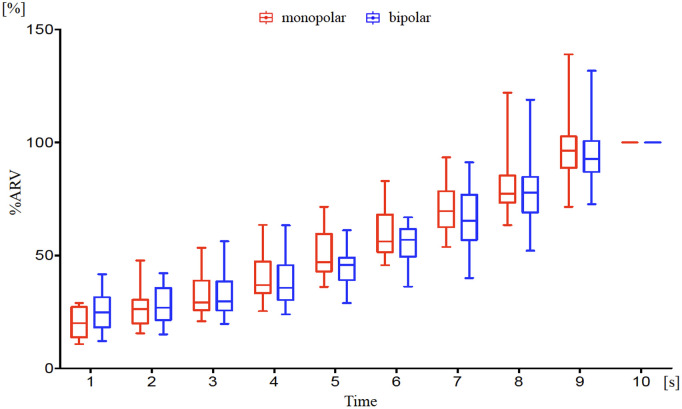
Temporal changes in % average rectified value (ARV) in monopolar and bipolar recording methods using multichannel surface electromyography. %ARV increased over time in both the monopolar (red) and bipolar (blue) recording methods. %ARV showed no significant differences between the recording methods in any of the intervals.

**Fig 4:**
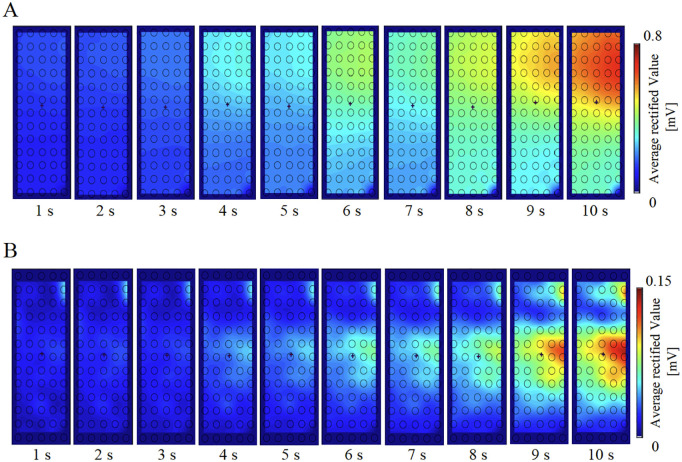
Representative ARV map for monopolar and bipolar recording methods in a participant over a 10-second period. Muscle activity increases with brighter colors (note that the lower right is blue due to the absence of one electrode). The participant exhibited higher muscle activity as contraction intensity increased over time. **(A)** With the monopolar recording method, muscle activity tended to increase in the middle to proximal medial regions as contraction intensity rose over time. **(B)** In contrast, the bipolar recording method showed a tendency for higher muscle activity in the middle medial regions.

**Fig 5:**
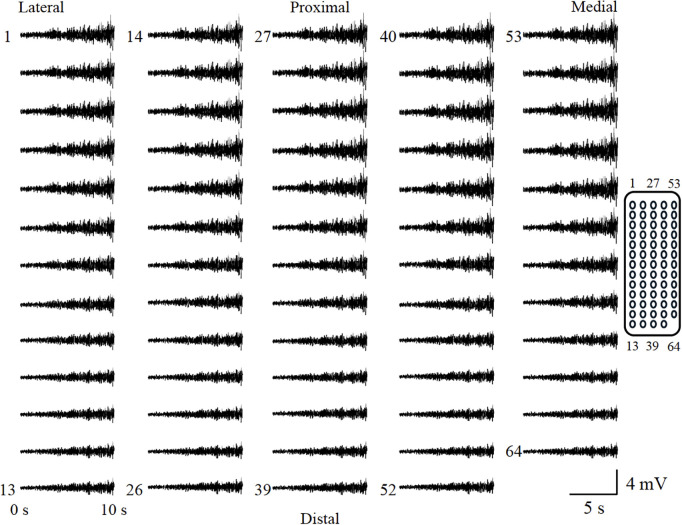
The representative raw waveforms from the monopolar recording method. Each waveform corresponds to the illustration on the right, and the amplitude increased with the rise in contraction intensity.

**Fig 6:**
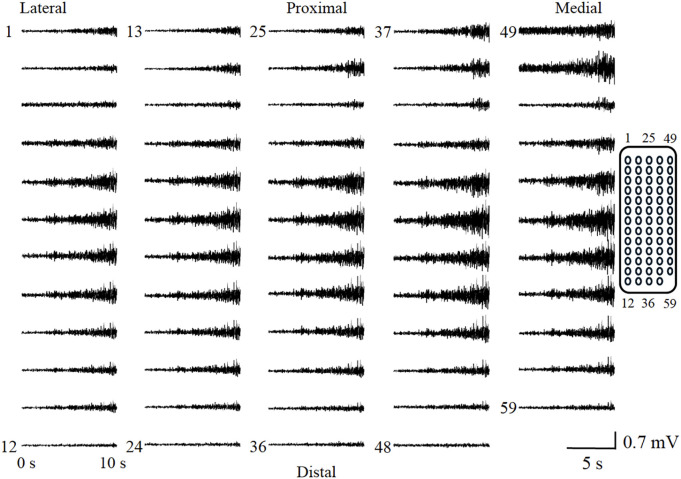
The representative raw waveforms from the bipolar recording method. Each waveform corresponds to the illustration on the right, and the amplitude increased with the rise in contraction intensity.

Figs 2–3 show the trends in the average values of %MVC and %ARV. Both %MVC and %ARV increased almost linearly from 0 to 10 seconds. No gender differences were observed in either %MVC or %ARV (Table 1). Additionally, comparisons of %ARV between monopolar and bipolar derivation methods showed no significant differences and were consistent (Fig 3). Next, representative ARV maps at each second from 0 to 10 seconds for a participant are shown in Fig 4. Muscle activity increased with contraction intensity, showing higher activity in the proximal medial region relative to the middle region with the monopolar recording method, and higher activity in the middle medial region using the bipolar recording method (Fig 4). The representative raw waveforms for each recording method are shown in Figs 5–6.

**Fig 7:**
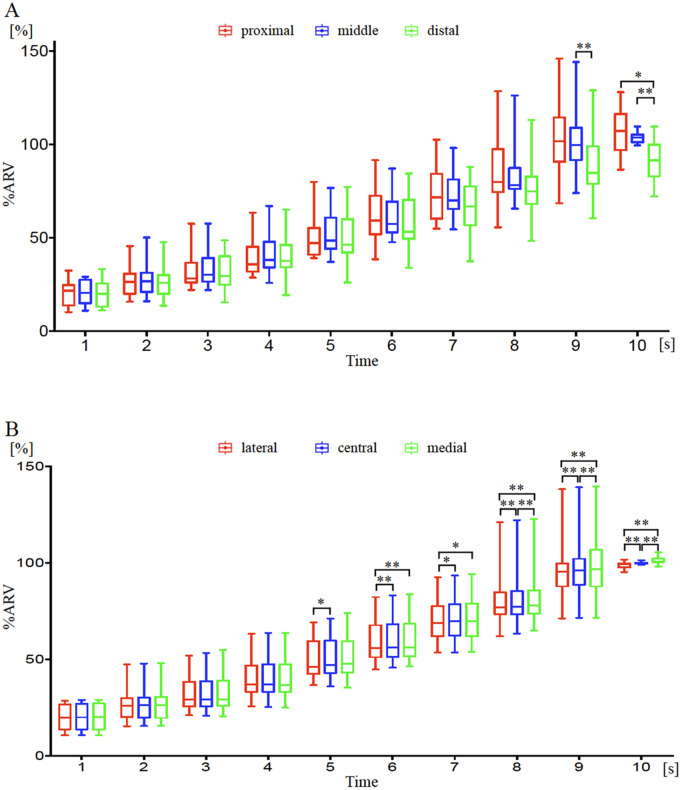
Temporal changes in %ARV over time by region using the monopolar recording method over a 10-second period. **(A)** As contraction intensity increased, muscle activity in the proximal (red) and middle (blue) regions was significantly higher than in the distal (green) region. **(B)** With increased contraction strength, muscle activity in the central (blue) and medial (green) regions was significantly higher than in the lateral (red) region. *p < 0.05, **p < 0.01.

**Fig 8:**
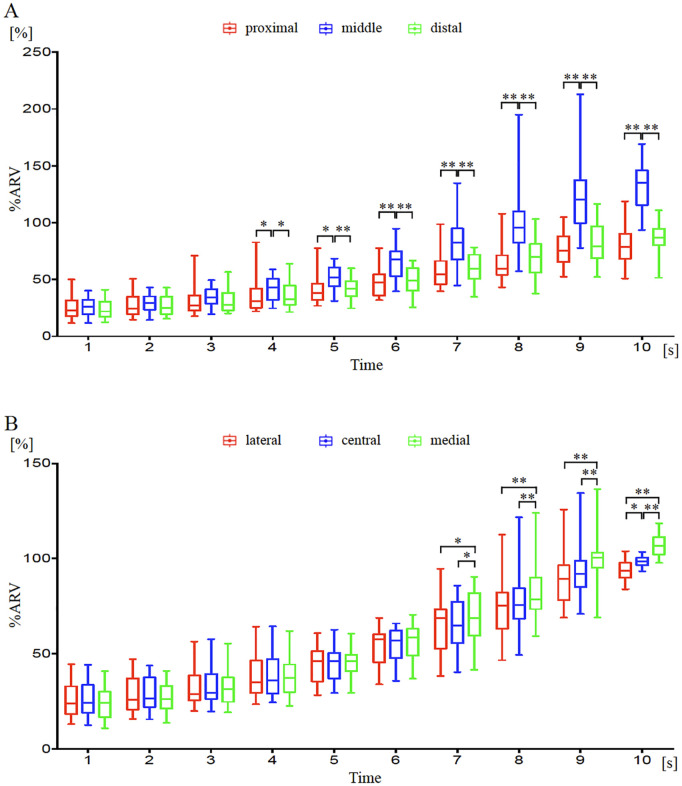
Temporal changes in %ARV over time by region using the bipolar recording method over a 10-second period. **(A)** As contraction intensity increased, muscle activity in the middle (blue) was significantly higher than in the proximal (red) or distal (green) regions. **(B)** With increased contraction strength, muscle activity in the central (blue) and medial (green) regions was significantly higher than in the lateral (red) region. *p < 0.05, **p < 0.01.

Figs 7–8 show the changes in %ARV in different regions with increasing muscle output using the monopolar and bipolar recording methods. Results from two-factor repeated measures ANOVA with correspondence showed that both interactions were significant (p < 0.01). In the comparison of %ARV divided into three segments of 4-5 rows each, significant main effects were observed at 9-10 seconds (p < 0.05) and at 4-10 seconds (p < 0.01). Regional differences at each time point in the monopolar recording method showed significant differences at 9-10 seconds between the middle and distal regions (p < 0.01), at 10 seconds between the proximal and distal regions (p < 0.05), and between the middle and distal regions (p < 0.01) (Fig 7A). In the bipolar recording method, significant differences were observed between the proximal and middle regions at 4-10 seconds (4-5 seconds: p < 0.05, 6-10 seconds: p < 0.01), and between the middle and distal regions at 4-10 seconds (4 seconds: p < 0.05, 5-10 seconds: p < 0.01) (Fig 8A). In the comparison of %ARV divided into three segments for every 1-2 columns, significant main effects were observed at 5-10 seconds (5, 7 seconds: p < 0.05; 6, 8-10 seconds: p < 0.01) and at 6-10 seconds (6-7 seconds: p < 0.05; 8-10 seconds: p < 0.01). Regional differences at each time point in the monopolar recording method showed significant differences at 5-10 seconds between the lateral and central regions (5, 7 seconds: p < 0.05; 6, 8-10 seconds: p < 0.01), at 6-10 seconds between the lateral and medial regions (7 seconds: p < 0.05; 6, 8-10 seconds: p < 0.01), and at 8-10 seconds between the central and medial regions (p < 0.01) (Fig 7B). In the bipolar recording method, significant differences were observed between the lateral and medial regions at 7-10 seconds (7 seconds: p < 0.05, 8-10 seconds: p < 0.01), between the central and medial regions at 7-10 seconds (7 seconds: p < 0.05, 8-10 seconds: p < 0.01), and between the lateral and central regions at 10 seconds (p < 0.05) (Fig 8B).

## Discussion

This study is the first to directly compare the distribution of muscle activity in the BB muscle during isometric contraction using multi-channel surface EMG with monopolar and bipolar recording methods. No significant differences were observed between the two recording methods when comparing overall normalized muscle activity across the muscle (Fig 3). These results align with findings from a previous study, which also reported similar normalized values for overall muscle activity in both recording methods. This consistency suggests that normalization helps minimize the influence of physiological and technical factors such as muscle mass and electrode placement [7]. In this study, we believe that normalization similarly contributed to the absence of significant differences in overall muscle activity between the recording methods. Conversely, when comparing different regions using %ARV, muscle activity increased from the central to proximal medial region in the monopolar method, while in the bipolar method, it increased in the central medial region as contraction strength increased (Figs 4, 7-8). The differences observed between the two recording methods in regional comparisons are most likely due to the characteristics of ARV, a common technique used to extract muscle activity from EMG signals. In the monopolar recording method, the recorded signals are directly analyzed into ARV. In contrast, the bipolar recording method involves first differentiating the signals recorded by the monopolar recording method before converting them into ARV. Since ARV presents results as absolute values for both methods, the bipolar recording method may show lower muscle activity in regions with small potential differences, even if the overall muscle potential is high. Moreover, if the electrodes are too close to each other, the potential difference may be insufficient, potentially reducing signal resolution [6]. The optimal interelectrode distance for bipolar derivation is generally recommended to be around 20 mm [13]. In multichannel surface EMG, the interelectrode distance may be too short, making it difficult to obtain adequate waveforms. Therefore, the monopolar recording method, which directly converts muscle potentials into muscle activity, is considered more suitable for comparing muscle activity distributions within the same muscle.

Although MRI and EMG assess fundamentally different physiological phenomena, with MRI evaluating muscle shortening and EMG capturing electrical activity, previous MRI studies have demonstrated that during elbow flexion, the degree of shortening in the BB muscle decreases in the distal region. This phenomenon is attributed to the mechanical constraints imposed by tendinous and aponeurotic structures, particularly as contraction intensity increases [14]. In contrast, in the middle and proximal regions, which lack tendons and aponeuroses, the shortening increases from the middle to proximal regions, leading to higher muscle activity compared to the distal region [14]. The motor point of the BB muscle is located at the midpoint of the reference line connecting the coracoid process and the lateral epicondyle of the humerus [15]. In this study, electrodes were placed such that the center of the electrode array aligned with the distal third of the line connecting the acromion and the cubital fossa. Therefore, it is presumed that the motor point is located slightly proximal to the center of the electrode placement. The innervation zone, where numerous neuromuscular junctions are concentrated, is primarily located in the middle region of the BB muscle [16] and shows higher muscle activity with the monopolar recording method [17]. Furthermore, as the innervation zone shifts proximally with increasing contraction intensity [18], muscle activity in the proximal region is expected to be higher than in the distal region. Additionally, the influence of the short head of the BB muscle leads to an increase in motor units in the medial part of the BB muscle, resulting in higher action potentials in this region [3]. These findings are consistent with the increased muscle activity from the middle to proximal medial regions observed with the monopolar recording method in this study.

There are several limitations in this study. The first is the impact of noise and crosstalk in the monopolar recording method [4,8]. To minimize this impact, we used isometric contraction tasks without joint movement, ensured adequate skin preparation, and visually confirmed the absence of noise in a resting state before starting the tasks. The comparison of normalized values between the monopolar and bipolar recording methods (Fig 3) showed a consistent trend with increasing contraction intensity. Therefore, we believe that comparing normalized values effectively minimized the effects of noise and crosstalk in the monopolar recording method. Second, only the BB muscle was examined, so it is not known if the findings are applicable to other muscles with different anatomical structures or functions. Third, the participants in this study were limited to healthy young adults. Future studies should explore older adults and individuals with motor function impairments, as well as examine multiple muscles, to provide a broader understanding of muscle activity distribution.

Nevertheless, the detailed insights provided by the monopolar recording method offer significant potential for practical applications. After excessive eccentric exercise, muscle activity in the proximal region of the BB muscle has been reported to significantly decrease [19]. Sustained uneven muscle activity can compromise joint stability and increase stress on the joints, potentially leading to injury [20]. Therefore, it is crucial to avoid overloading the proximal region and to maintain uniform muscle activity to prevent injuries. The monopolar recording method using multichannel surface EMG, as employed in this study, is considered useful for determining the optimal load for muscle strengthening exercises in the BB muscle.

## Conclusion

As contraction intensity increased during isometric contraction, muscle activity in the BB muscle was significantly higher in the middle to proximal medial region with the monopolar recording method and in the middle medial region with the bipolar recording method. The monopolar recording method using multichannel surface EMG may be useful for understanding the distribution of muscle activity within the BB muscle because the results were consistent with previous reports regarding the increased shortening rate of the BB muscle from the middle to the proximal area and the migration of the innervation zone.

## Supporting information

S1 Data‌‌ Individual raw data used in the analysis. (XLSX)
